# Understanding Light Harvesting in Radial Junction Amorphous Silicon Thin Film Solar Cells

**DOI:** 10.1038/srep04357

**Published:** 2014-03-12

**Authors:** Linwei Yu, Soumyadeep Misra, Junzhuan Wang, Shengyi Qian, Martin Foldyna, Jun Xu, Yi Shi, Erik Johnson, Pere Roca i Cabarrocas

**Affiliations:** 1 School of Electronics Science and Engineering/National Laboratory of Solid State Microstructures, Nanjing University, 210093, Nanjing, China; 2 Laboratoire de Physique des Interfaces et Couches Minces (LPICM), Ecole Polytechnique/CNRS, 91128 Palaiseau, France

## Abstract

The radial junction (RJ) architecture has proven beneficial for the design of a new generation of high performance thin film photovoltaics. We herein carry out a comprehensive modeling of the light in-coupling, propagation and absorption profile within RJ thin film cells based on an accurate set of material properties extracted from spectroscopic ellipsometry measurements. This has enabled us to understand and evaluate the impact of varying several key parameters on the light harvesting in radially formed thin film solar cells. We found that the resonance mode absorption and antenna-like light in-coupling behavior in the RJ cell cavity can lead to a unique absorption distribution in the absorber that is very different from the situation expected in a planar thin film cell, and that has to be taken into account in the design of high performance RJ thin film solar cells. When compared to the experimental EQE response of real RJ solar cells, this modeling also provides an insightful and powerful tool to resolve the wavelength-dependent contributions arising from individual RJ units and/or from strong light trapping due to the presence of the RJ cell array.

Radial junction (RJ) solar cells^1^,^2^ have been fabricated by deposition on top of a matrix of Si nanowires (SiNWs), prepared either by template-assisted etching into Si wafers^3^,^4^,^5^ or by self-assembled growth on low-cost substrates^6^,^7^,^8^,^9^. Prototype devices of this kind indeed demonstrate superior light harvesting performance, achieved due to *a* *long* *optical absorption length* while using only *a* *thin absorber/or junction layer*^4^,^5^,^10^,^11^. Though several recent works have addressed the full-field simulation of an array of Si nanowire/rods or radial *p-n* junctions^12^,^13^,^14^, the light in-coupling and absorption profile in a more sophisticated and complex multilayer *p-i-n* thin film solar cell structure, as well their implications for device performance, remain largely unexplored. Within a coaxial RJ thin film solar cell, a sequence of layers of materials with distinctive optical properties are deposited around individual SiNW cores^7^,^9^,^15^,^16^. In such a 1D cavity-like composite, photonic and resonant mode-matching effects in the RJ units via optical antenna coupling or leaky-mode-resonance^13^,^17^,^18^,^19^ can play an important role in determining light absorption and scattering. These phenomena have been observed experimentally in RJ solar cells fabricated over individual upright^20^ or surface-lying^15^ nanowires. How this resonant absorption or scattering will affect the light harvesting behavior in a coaxial *p-i-n* junction thin film solar cell remains an interesting aspect to address. Furthermore, in pursuit of an optimal light harvesting configuration but that can be easily electrically contacted, a “forest” of nanowires standing upright on a substrate surface could provide an ideal and realistic architecture for making and contacting large areas of RJ solar cells. Therefore, a comprehensive and accurate description of the light in-coupling, propagation and absorption profile within the coaxial multilayer structure will be very helpful to shed more light upon the critical issues, and to design a new generation of high performance RJ thin film solar cells.

In this study, we focus on a coaxial RJ solar cell, as illustrated in Fig. 1(a), fabricated around SiNWs grown via a vapor-liquid-solid (VLS) mechanism catalyzed by a low-melting-point metal such as tin (Sn)^21^,^22^,^23^,^24^. Low-melting-point metal catalysts, including Sn, In, Ga and Bi, allow one to achieve the low-temperature growth (down to 240°C) of a well-defined matrix of SiNWs^22^,^25^. This low-temperature process is compatible with becoming a mature thin film technology, as well as having the benefit that the metal remnants can be cleaned off easily by a simple H_2_ plasma etching without the need to break vacuum for *ex-situ* chemical etching/cleaning^7^,^9^,^24^. Figure 1(b) presents a scanning electron microscopy (SEM) view of such a structure. Our recent progress using this structure has led to the demonstration of high performance radial p-i-n junction amorphous silicon (a-Si:H) thin film solar cells with a power conversion efficiency of 8.14%^7^, all fabricated in a conventional plasma enhanced chemical vapor deposition (PECVD) system. Our goal in this work is therefore to establish a comprehensive optical model for a coaxially-deployed multilayer RJ thin film solar cell, in order to develop critical clues and a new strategy for optimizing such devices.

## Experimental Description

**Figure 1:**
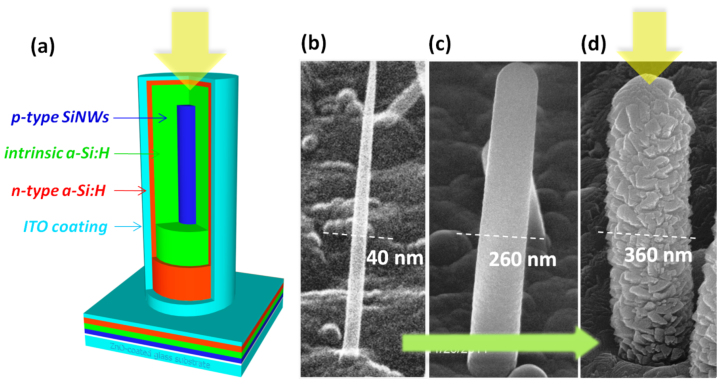
(a) Schematic illustrating the coaxial multilayer structure of a radial junction solar cell unit; (b), (c) and (d) show respectively the SEM images of a single SiNW, a p-i-n RJ cell and an ITO coated RJ cell.

The RJ solar cells were deposited on a matrix of SiNWs grown on top of ZnO:Al coated Corning glass (Cg) substrates. The Sn catalyst droplets were formed *in situ* by a H_2_ plasma treatment of a 2 nm thick (nominal) Sn layer evaporated on the ZnO:Al substrate. SiNWs were grown and doped p-type by flowing a mixture of silane (SiH_4_) and H_2_-diluted TMB [(CH_3_)_3_B] during the plasma-enhanced VLS growth. The detailed fabrication procedure and deposition parameters are available in our previous work^7^. As one can see in Fig. 1(b), the typical SiNW features a gradually tapering shape with a length of *L_w_* ~ 1.7 μm, a diameter of ~40 nm in the middle and a sharp tip of <20 nm. As the next step, an intrinsic a-Si:H layer (typically ~100 nm thick) and an n-type doped a-Si:H emitter layer (of ~10 nm) were coated consecutively over the SiNWs, followed by a sputtered indium-tin-oxide (ITO) top contact (T_ito_ ~ 50 nm) over the RJ cell. The RJ cells without and with the TCO coating layers are shown in Fig. 1(c) and 1(d), respectively, while an internal multilayer structure consisting of p-type SiNW-core (*p*)/intrinsic a-Si:H (*i*)/n-type a-Si:H (*n*)/ITO contact is depicted in Fig. 1(a).

## Formulation and Simulations

To carry out an accurate simulation, the optical dispersion n-k curves of each material have been extracted from the modeling of spectroscopic ellipsometry measurements on co-deposited planar thin films (as shown in S.1 in Supplemental Materials). In this RJ simulation, we assume the SiNW core is represented by a cylinder of radius *R_w_* = 20 nm and with lengths (*L_w_*) from 0.6 μm to 2.4 μm. The intrinsic a-Si:H layer thickness (*T_i_*) has been varied from 20 nm to 140 nm, while the thickness of the n-type a-Si:H layer and ITO layers are fixed to be typical values of *T_n_* = 10 nm and *T_ito_* = 50 nm, respectively, consistent with what is seen in Fig. 1(b) to 1(d). In our simulation, a single radial cell is placed at the center of a square simulation box (1.5 μm × 1.5 μm) on top of a glass substrate. Polarized light propagating along the y-axis originates from the top plane. Dissipative scattering conditions are applied to the entirety of the wall and bottom boundaries. The finite element solver module in COMSOL MULTIPHYSICS was used to carry out the structural modeling and optical simulation.

**Figure 2:**
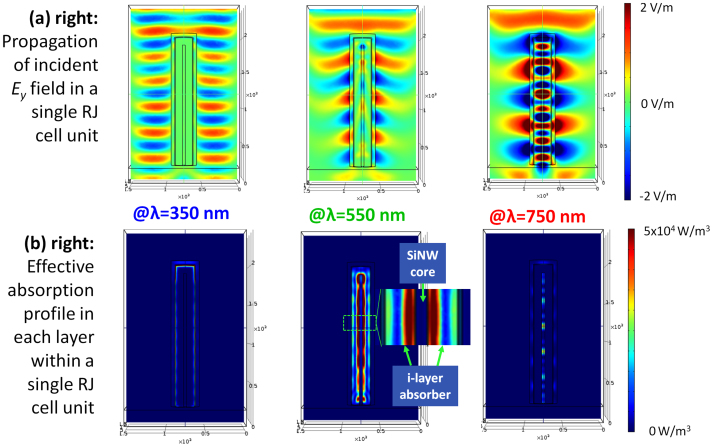
(a) Upper panels show the simulated electrical field distribution (*E_y_*) through a single RJ cell at different photon wavelengths, while the lower panels (b) present their corresponding absorption profile within each material layer in the multilayer RJ structure.

In contrast to the situation of radial p-n junction solar cells fabricated on Si wafers^3^,^26^, the incident light has to go through first the top TCO and then the n-type emitter layers before arriving at the inner intrinsic a-Si:H absorber in the radial p-i-n junction thin film solar cell. As a consequence, assessing the optical propagation and losses within the coaxial radial configuration is a critical issue. In the upper panel in Fig. 2(a), we show the *E_y_*-field distribution in a SiNW RJ solar cell unit under different incident wavelengths of λ = 350 nm, 550 nm and 750 nm, with a SiNW length of *L_w_* = 1.7 μm and i-layer thickness of *T_i_* = 100 nm. At λ = 350 nm (with high energy photons), the incident light is screened by the outer ITO and n-type emitter layers, with only a small fraction reaching the buried absorber i-layer. At long wavelengths (λ = 550 nm to 750 nm), the incident light propagates deeper into the RJ, and can be better coupled into the cavity-like RJ cell, which starts to behave particularly like a waveguide or antenna at λ = 750 nm.

Based on these field distribution results, the local power dissipation - that is the local absorption occurring within the RJ multilayer - is calculated according to 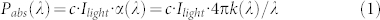
where *I_light_* is the local electromagnetic energy intensity, *c* is the speed of light in vacuum, *α*(*λ*) and *k*(*λ*) are the absorption coefficient and the imaginary part of the complex refractive index 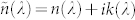. The corresponding power absorption mappings in the RJ cell structure are calculated and presented in the lower panel of Fig. 2(b). The absorption of high energy photons (*λ* = 350 nm) mostly takes place within the outer ITO and n-type emitter layers, with little absorption occuring in the PV active inner intrinsic a-Si:H layer. At *λ* = 550 nm, the inner i-layer becomes the dominant zone for light harvesting, while the absorption in the c-SiNW core remains still very weak, in spite of a strong field within the core region as seen in the corresponding *E_y_* field distribution in Fig. 2(a). This is because at λ = 550 nm, the absorption coefficient of a-Si:H is almost two orders of magnitude higher than that of c-Si (typically *α_a-Si:H_/α_c-Si_* ~ 10^2^). At the even longer wavelength of λ = 750 nm, when the incident photon energy approaches the optical bandgap of the a-Si:H absorber matrix, the incident light has been strongly confined within the RJ cell. However, the effective absorption realized within the intrinsic a-Si:H layer drops dramatically, see Fig. 2(b), while most incident photons get absorbed in the center SiNW, which is considered as a dead zone because the high doping level in the SiNW core electrode.

**Figure 3:**
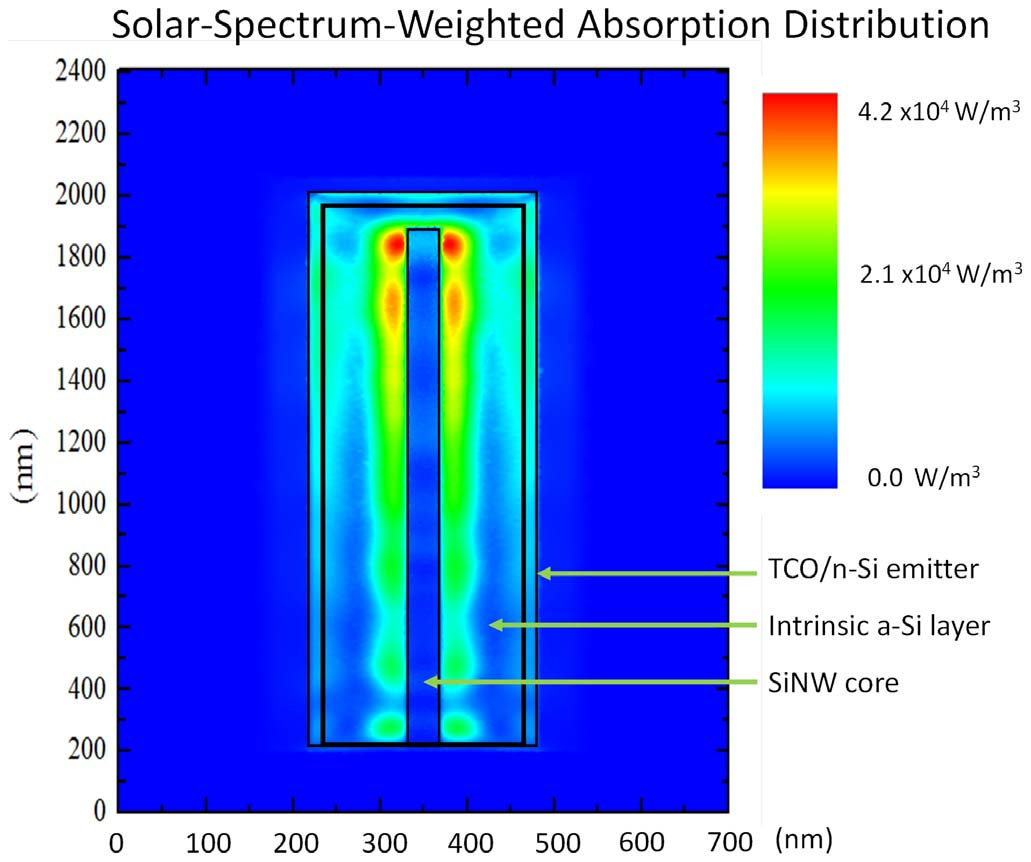
Solar-spectrum-weighted absorption distribution within the RJ solar cell unit, over a spectrum through λ = 300 nm to 800 nm.

Close examination of the absorption mapping at λ = 550 nm shown in Fig. 2(b) reveals yet more intriguing features of the absorption profile within a single RJ cell. For example, the highest absorption regions in the i-layer are actually located very close to the inner i-layer/SiNW interface, as witnessed in the enlarged inset in Fig. 2(b) for λ = 550 nm, instead of at the outer emitter/i-layer interface, as one would expect in a planar p-i-n thin film solar cell. In Supplemental Material S2, absorption profiles within the RJ solar cell unit at varying incident wavelengths running from 300 nm to 800 nm are compiled and displayed in an animation clip. We found that this feature is a rather common characteristic for wavelengths longer than roughly λ > 475 nm. Furthermore, to give a quantitative estimation of PV performance, we also calculate a solar-spectrum-weighted absorption profile *P_av_* within a RJ solar cell unit, as shown in Fig. 3, defined as 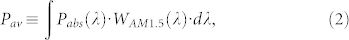
where *W_AM_*_1.5_(*λ*) is the weighting factor derived from the standard AM1.5 solar mass spectrum with 
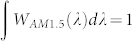
 when integrating over λ = 300 nm to 800 nm (the most relevant spectrum range for a-Si:H absorption). Note that, the *x*-axis to *y*-axis aspect ratio in Fig. 3 has been intentionally enlarged to 3:1 to deliver a better view of the low absorption density variation in the lateral direction. It is clear that the absorption intensity profile shows its highest absorption zones in the vicinity of the inner i-layer/SiNW core interface, instead of the outer emitter/i-layer interface. This phenomenon indicates that the incident light has been coupled effectively into the RJ structure, following the field distributions of the resonant propagating modes within the 1D RJ cell cavity. We note that this scenario is strikingly different to the situation in planar p-i-n junction a-Si:H cells, where the highest absorption zones are always located at the outer emitter/i-layer interface, and therefore a p-type emitter layer is always preferable to shorten the collection distance of photo-generated holes (considering its lower mobility in the a-Si:H matrix). Our finding here reveals however a quite different situation, in that a higher concentration of photo-carriers are generated in close proximity to the inner interface and thus a p-type SiNW core should become more favorable. This is indeed an important design constraint for optimizing RJ a-Si:H thin film solar cells.

**Figure 4:**
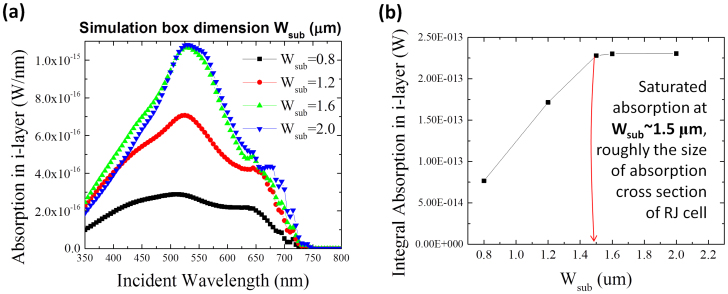
(a) Effective absorbed power within the i-layer at specific photon wavelengths and as a function of different simulation box dimension (*W_sub_*); (b) evolution of the integrated absorption within the i-layer.

Furthermore, by integrating the absorption in each material layer of the RJ solar cell, we are able to calculate the effective absorption power *P_eff_*(λ) within the absorber i-layer as a function of photon wavelength ranging from 300 nm to 800 nm. As we can see in Fig. 2(a), the RJ cell can harness the incident light over a cross sectional area much larger than its physical diameter, that is, the light can be effectively collected into the 1D RJ cell (like a waveguide or an antenna)^20^,^27^. This becomes more prominent for the incidence at long wavelengths. So, when we hope to address the absorption potential of an individual RJ, the finite lateral dimension of the square simulation box *W_sub_* should be large enough to exclude (or at least minimize) the boundary effects. To be sure of this point, we first calculate the *P_eff_*(*λ*) curves with different box dimensions, extending from *W_sub_* = 0.8 μm to 2.2 μm. Their corresponding *P_eff_*(*λ*) curves and overall absorption integral (*P_oa_*) from λ = 300 nm to 800 nm are presented in Fig. 4(a) and 4(b), respectively. We found that the overall integral increases gradually with *W_sub_* before saturating at *W_sub_* = 1.5 μm and above, indicating that from this point on the RJ cell becomes basically optically isolated within the wavelength range investigated here. Based on this observation, we define an effective overall absorption cross-section area of 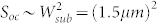 for such a single RJ a-Si:H cell, and extract an enlargement factor (*F*) of *S_oc_* over its physical cross section area (*S_RJ_*) of the RJ cell as 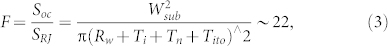
From this point on, we choose to fix the simulation box dimension to *W_sub_* = 1.5 μm in the following simulations unless otherwise specified.

**Figure 5:**
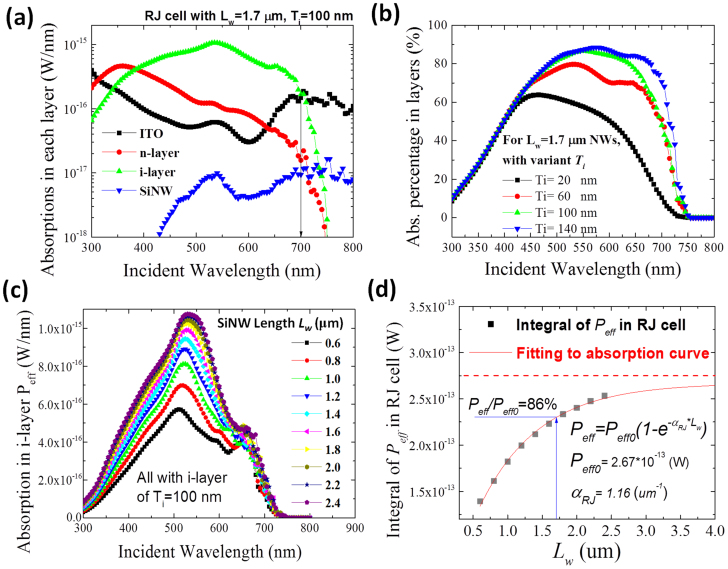
(a) Absorbed power broken down by material layer, at varying incident photon wavelength for a RJ cell with SiNW length of *L_w_* = 1.7 μm and i-layer thickness of *T_i_* = 100 nm. (b) Percentage of effective absorption in the i-layer relative to total absorption in the RJ multilayer structure. (c) Evolution of the absorption spectra in the active i-layer with different Si nanowire lengths from *L_w_* = 0.6 um to 1.7 μm. (d) full spectrum integrated absorption in i-layer as a function of SiNW length. This trend is then fitted by Lambert-Beer law as marked by the red line.

To understand the optical losses and effective absorption within the RJ cell, let us look at the absorption broken down by layer in Fig. 5(a), for a typical RJ a-Si:H solar cell unit, as seen in Fig. 1(b)–1(d) with *L_w_* = 1.7 μm, *T_i_* = 100 nm and *W_sub_* = 1.5 μm. The absorbed power is plotted the on y-axis in log-scale. It is clear that a majority of the absorption losses at short photon wavelengths (below 400 nm) are caused by the outer ITO and n-type a-Si:H emitter layers. In particular, the n-type a-Si:H accounts for quite a large portion of the overall losses, emphasizing the need to adopt a wider band-gap emitter, for instance doped a-SiC^28^ or μc-SiO_x_^29^,^30^ layers. In the long wavelength region, the absorption losses in ITO contact become dominant, while the absorption in both i-layer and n-type emitter drop rapidly when approaching the bandgap of a-Si:H. Figure 4(b) shows the absorption occurring in the i-layer as a percentage of the total absorption in the RJ cell, shown for i-layer thicknesses (*T_i_*) ranging from 20 nm to 140 nm. As expected, a thicker i-layer can boost absorption for longer wavelengths λ > 450 nm, but this tends saturate for *T_i_* > 100 nm. Further increasing the thickness up to 140 nm leads to only marginal gain, and only for wavelengths >700 nm when the absorption in a-Si:H becomes very weak. It is noteworthy that the i-layer thickness of 100 ~ 140 nm used here is only 1/3 ~ 1/2 of the typical a-Si:H i-layer thicknesses used in optimized planar p-i-n junction a-Si:H solar cell (280 or 300 nm to balance absorption and collection). A thinner i-layer establishes a stronger electric field in the i-layer, facilitating photo-carrier separation and collection, and thus helps to improve solar cell efficiency and stability^28^,^31^,^32^.

Another important parameter of the RJ thin film solar cell is the length *L_w_* of SiNW, which represents a new dimension to adjust in the 3D RJ architecture, in contrast to their planar counterparts. In Fig. 5(c), we plot *P_eff_* as a function of the length of SiNWs, ranging from 0.6 μm to 2.4 μm. With increasing *L_w_*, there is an overall enhancement of the i-layer absorption for wavelengths λ < 650 nm, while for the longer wavelengths the absorption enhancement is rather marginal. The integral of *P_eff_* from 300 nm to 800 nm has been calculated and shown in Fig. 5(d), where we see first a fast increase of *P_eff_* absorption with the SiNW length, then followed by a clear saturation trend. At λ > 650 nm, the absorption coefficient of a-Si:H matrix drops quickly and the absorption of incident light is dominated by the ITO layer and c-SiNW core as shown in Fig. 5(a), as well as by the dissipation on the damping boundary walls. To quantify the overall light harvesting behavior as a function of the length of RJ cell, we fit the data in Fig. 5(d) following the formulation of *Lambert-Beer* law, which can be written as 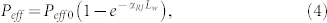
and extract a saturation absorption of *P_eff_*_0_ = 2.67 × 10^−12^ *W* for *L_w_* → ∞, as shown in Fig. 5(d) by a dashed horizontal line, and an overall *pseudo-absorption coefficient* for such a single RJ cell to be *α_RJ_* = 1.16/*μm* (or 1.16 × 10^4^/cm). According to this prediction, a RJ cell with a 1.7 μm long SiNW core already absorbs ~86% of the light absorbed by an infinitely long RJ cell. This provides a very useful and practical guide to evaluate the benefit of growing long SiNWs, against the need to keep the SiNW short to facilitate the conformal coating of closely spaced SiNWs.

**Figure 6:**
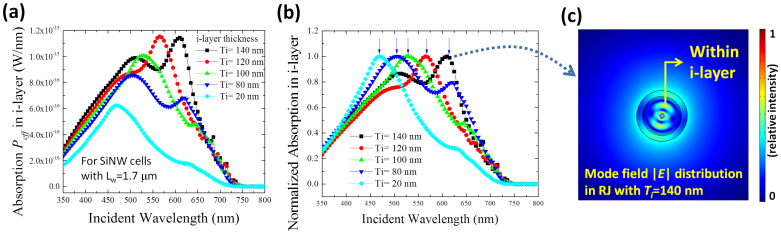
(a) Absorption spectra in active i-layer while varying i-layer thickness (*T_i_*) from 20 nm to 140 nm, (b) full spectrum integrated absorption in i-layer as a function of *T_i_*. (c) field distribution of mode in the RJ cross section with *T_i_* = 140 nm.

Furthermore, the impact of the i-layer thickness (*T_i_*) on the total light harvesting behavior has been studied. In this simulation, we fix the length of the SiNW at *L_w_* = 1.7 um. The calculated absorption curves for different *T_i_* ranging from 20 nm to 140 nm are shown in Fig. 6(a). With the increase of i-layer thickness from 20 nm to 80 nm, the effective light absorption *P_eff_* enhances greatly over the full spectrum, particularly in the range from 450 nm to 650 nm. Interestingly, upon all the *P_eff_* curves, we observe superimposed absorption peaks that seem to shift systematically with i-layer thickness. To clarify this trend, we have normalized all these curves to their maxima in Fig. 6(b) to reveal a clear blue-shifting of the absorption peaks with decreasing i-layer thickness. To understand this point, we have carried out a 2D mode analysis on the cross section of a coaxial RJ cell with an i-layer thickness of *T_i_* = 140 nm. At the wavelength of the absorption peak at λ = 614 nm, we search for the first 6 propagation modes in the RJ cavity and then integrate their individual contributions to the power absorbed within the i-layer region. We found that the highest contribution comes from a pair of propagation modes with mutually orthogonal field distribution patterns, one of which is shown in Fig. 6(c). As we can see, for this cavity mode, the field distribution is concentrated in the annular i-layer, resulting in a maximal useful absorption in the RJ cell. On the contrary, other propagation modes found in the RJ cavity (not shown here) display effective wave-guiding around the RJ cavity, but have their field intensity maxima falling out of the intrinsic absorber layer (either in the outer ITO/n-type layer or concentrated into the highly doped p-type SiNW core). Such distributions lead to a much lower useful absorption for the radial p-i-n junction thin film solar cell. This resonant-mode-selection feature/or criterion is indeed a unique aspect in the coaxial radial p-i-n cell, as compared to the situation in a simple radial p-n junction solar cells.

**Figure 7:**
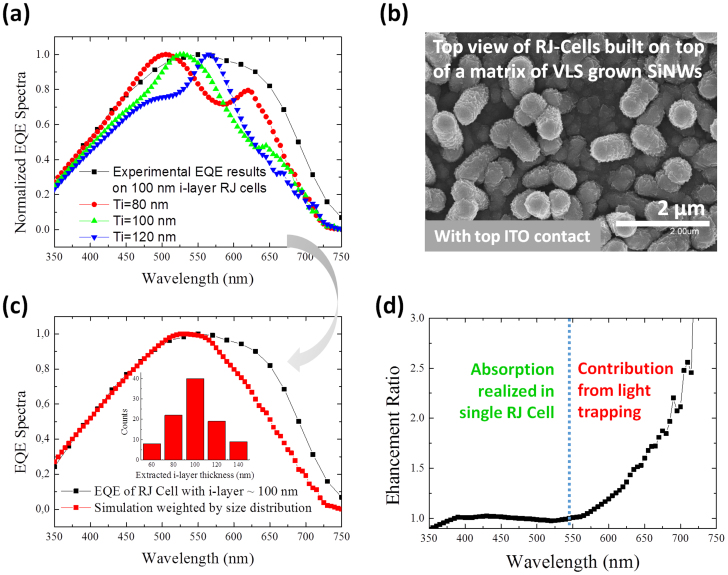
(a) Normalized experimental EQE spectrum (black square-line) measured for RJ cells on VLS-grown SiNWs (example SEM image in (b)), compared to the normalized calculated absorption curves for *T_i_* = 80 nm, 100 nm and 120 nm. (c) Normalized experimental EQE and averaged simulated absorption curves weighted according to their appearance statistics. (d) Spectrum of the absorption enhancement ratio observed for an array of RJ cells (with strong light trapping effect) over that of a single RJ cell, as extracted from (c).

Finally, assuming that the absorption of one incident photon (with energy higher than the bandgap of a-Si:H) generates only one electron-hole pair and a rapid thermalization of the hot photo-carriers occurs, the absorption *P_eff_* curve should correspond/or be proportional to the external quantum efficiency of a single RJ cell (excluding recombination issues). We therefore compare, in Fig. 7(a), the normalized *P_eff_* curves with i-layer thickness of 80 nm (red), 100 nm (green) and 120 nm (blue) to the normalized experimental EQE spectrum (black), measured on a real RJ cell array shown in the top-view SEM image of Fig. 7(b). The i-layer thickness of the RJ cell array is around 100 nm with a statistical spread as shown in the inset of Fig. 7(c). As a consequence, though there is a specific resonant peak position for each i-layer thickness, the weighted-average response curve becomes rather smooth and featureless. As we can see in Fig. 7(c), the agreement between the simulated overall absorption curve and the actual normalized EQE curve over the short-wavelengths (from λ = 300 nm to 550 nm) is indeed excellent, indicating that the light harvesting of short-wavelength photons can be mostly assigned to the separately contribution from individual RJ cells. In other words, the short-wavelength photons have been effectively harvested during their first pass through the radial *p-i-n* junction, with basically no contribution from the light trapping among neighboring RJ cells. At longer wavelengths, we notice a higher EQE response was measured for the actual “forest” of RJ solar cells, compared to the simulated response for a single RJ solar cell. This can be seen more clearly in Fig. 7(d), where the enhancement ratio of the normalized experimental EQE over the absorption response of a single RJ cell is plotted as a function of incident wavelength. We note that the enhancement factor is close to unity for wavelengths below <550 nm, but scales up monotonically with the increase of the wavelength.

This observation implies that the contribution of light trapping arising from multiple scattering events among individual RJ units is indeed wavelength-dependent. This is reasonable if we recall the fact that at long wavelengths, the absorption coefficient of a-Si:H absorber drops rapidly, and therefore allows multiple passes of the incident photons through the RJ cell units before being absorbed. Therefore, the benefit of strong light trapping gradually manifests itself at longer incident wavelengths. Alternatively, the greater experimental long-wavelength EQE can also be explained by the overlapping of the absorption cross-sections of neighboring RJ cells within a dense RJ forest as seen in Fig. 7(b). Though other factors or geometrical considerations (for example the somewhat random orientation of the VLS-grown Si nanowires on glass) could also be responsible for this behavior and need to be better understood in future studies, we emphasize that this comparison provides a unique perspective, emphasizing the relative importance of the light trapping effect in a matrix of RJ cells compared with the impact of the geometry of individual RJ cells.

## Conclusion

In summary, we have presented a comprehensive optical model of a single radial junction a-Si:H thin film solar cell, which incorporates a complete set of accurate optical material properties in each layer. Our simulations have been able to reveal the detailed light in-coupling, propagation and absorption profile in the complex coaxial multilayer solar cell structure, as well as the impact of varying a set of critical geometrical parameters. We also investigate the impact of photonic effect/resonant-mode enhanced absorption within the quasi-1D cavity radial junction cells, and NW design criteria for achieving maximal light absorption. These simulation results are further compared to experimental results on radial junction a-Si:H thin film solar cells fabricated on VLS-grown SiNWs. We note that the findings developed herein for RJ a-Si:H thin film solar cells are, in principle, applicable to radial junction solar cells fabricated by other techniques in different material systems, and therefore could provide insightful guidelines for structural optimization and improved performance.

## Author Contributions

L.Y., S.M., J.W. and P.R. designed/conducted the experiments and simulations, wrote the main manuscript text and prepared the figures. S.Q., M.F., J.X., Y.S. and E.J. have participated in analyzing the data and reviewing this manuscript.

## Supplementary Material

Supplementary Information Supplementary Materials

Supplementary Information Supplemental gif clip S-2
