# Supplementary material for: Understanding Light Harvesting in Radial Junction Amorphous Silicon Thin Film Solar Cells
Source: Sci Rep. 2014 Mar 12;4:4357. doi: 10.1038/srep04357 (PMC3950579; doi:10.1038/srep04357)
Supplement: Supplementary Information — Supplementary Materials [file srep04357-s1.pdf]

Supplemental Materials for

Understanding Light Harvesting in Radial Junction Amorphous Silicon Thin Film Solar Cells

Linwei Yu[1,2*], Soumyadeep Misra[2], Junzhuan Wang[1], Shengyi Qian[1], Martin Foldyna[2], Jun Xu,[1] Yi Shi,[1] Erik Johnson[2], and Pere Roca i Cabarrocas[2*]

[1] *School of Electronics Science and Engineering/National Laboratory of Solid State Microstructures,*

Nanjing University, 210093, Nanjing, China

[2] *Laboratoire de Physique des Interfaces et Couches Minces (LPICM), Ecole Polytechnique/CNRS, 91128 Palaiseau, France*

[1] Email: yulinwei@nju.edu.cn, pere.roca@polytechnique.edu

Supplemental Materials S.1

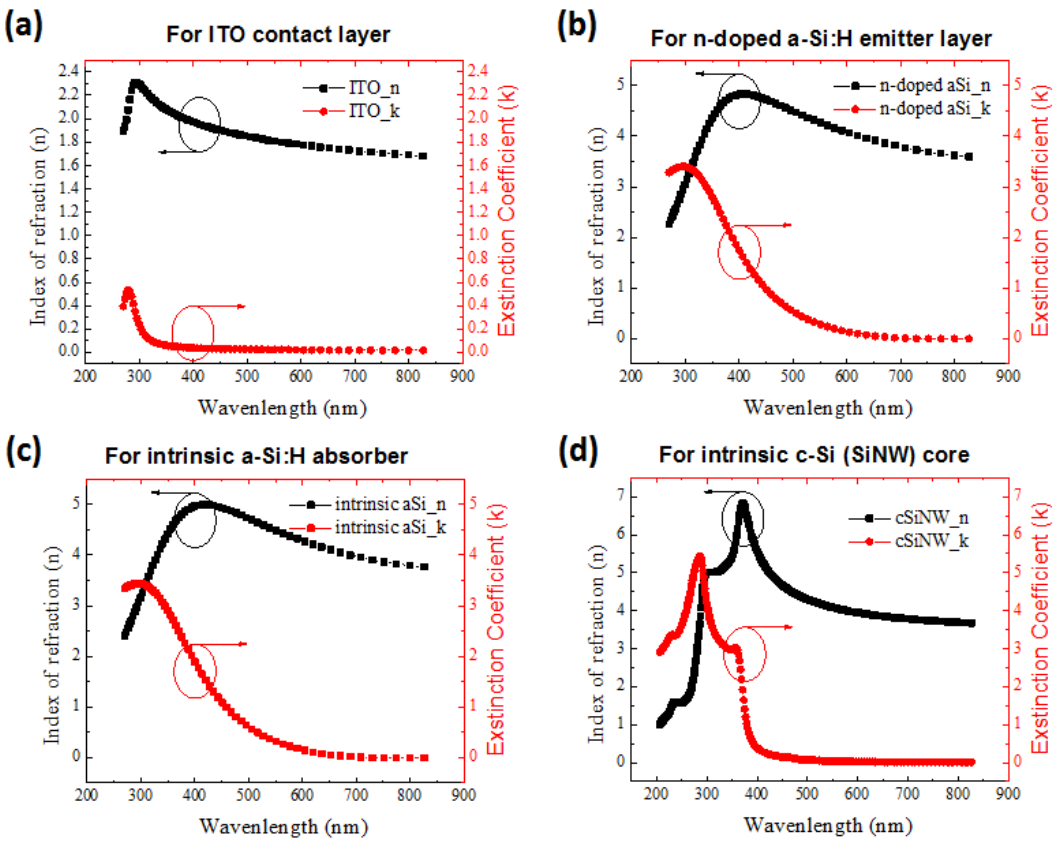

S. 1

S1: (a) – (d) show the n-k curves of the ITO, n-emitter a-Si:H, intrinsic a-Si:H and c-Si (SiNW core) adopted in the RJ solar cell simulations, extracted from/determined by spectroscopic ellipsometry characterizations on corresponding co-deposited thin film materials.

Supplemental Materials S.2

(See attached GIF file for animation)

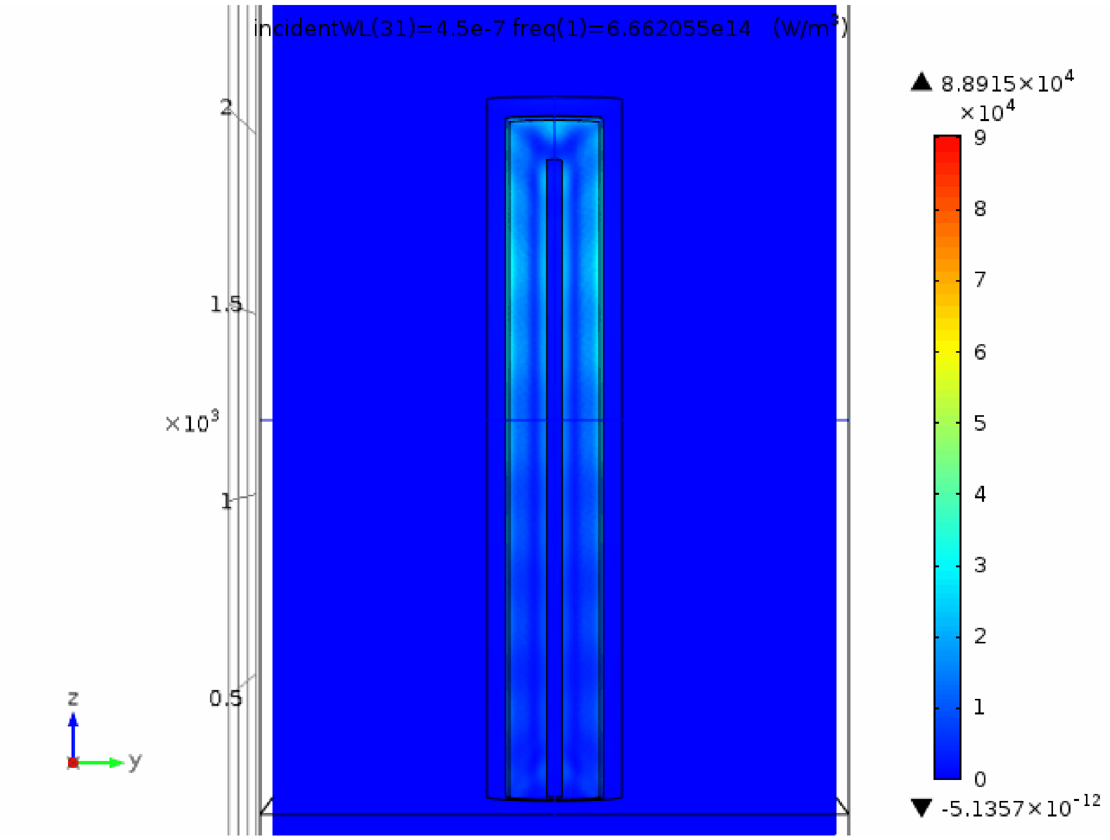

S. 2 an animation clip (See attached animation file in GIF format) that shows the absorption profile within the RJ unit when the incident wavelength running through λ=450 nm to 800 nm.
