# Supplementary figures and images for: Understanding Light Harvesting in Radial Junction Amorphous Silicon Thin Film Solar Cells
Source: Sci Rep. 2014 Mar 12;4:4357. doi: 10.1038/srep04357 (PMC3950579; doi:10.1038/srep04357)

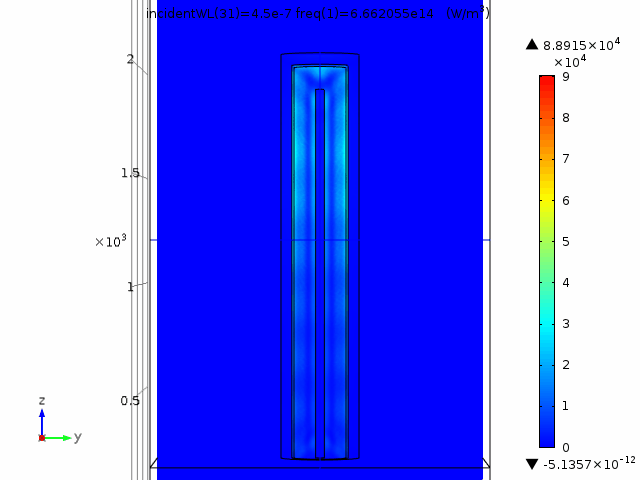

Supplement: Supplementary Information — Supplemental gif clip S-2 [file srep04357-s2.gif]
